# Characterization and identification of charcoal of inedible Kerandang fish (*Channa pleurophthalmus* Blkr) body parts and potential antiallergenic properties

**DOI:** 10.14202/vetworld.2020.1480-1486

**Published:** 2020-07-30

**Authors:** Aryani Aryani, Eddy Suprayitno, Bambang Budi Sasmito, Hardoko Hardoko

**Affiliations:** 1 Doctoral Program, Faculty of Fisheries and Marine Sciences, Brawijaya University, Malang 65145, East Java, Indonesia; 2 Department of Fisheries Product Technology, Faculty of Fisheries and Marine Sciences, Brawijaya University, Malang 65145, East Java, Indonesia

**Keywords:** anti-allergy, charcoal, hyaluronidase, Kerandang fish

## Abstract

**Background and Aim:**

The study about the antiallergenic properties of inedible fish body parts is still limited. Therefore, this study aimed to characterize the charcoal from the body parts of Kerandang fish (*Channa pleurophthalmus* Blkr) and identify its antiallergenic properties.

**Materials and Methods:**

This study used some non-edible body parts extracted from the Kerandang fish (i.e., the scalp, scales, and dorsal, pectoral, ventral, anal, and caudal fins) using a maceration method with different solvents (ethanol, ethyl acetate, and chloroform). The identification of active compounds in the extract was carried out using liquid chromatography–high-resolution mass spectrometry (LC-HRMS) analysis, while the antihyaluronidase activity was determined using the antihyaluronidase test. The highest charcoal antihyaluronidase activity-extract was applied to ovalbumin-induced mice for 7 days with various doses (10, 15, and 20 mg/kg). The specific immunoglobulin E (IgE) was measured using enzyme-linked immunosorbent assay on day 8.

**Results:**

Our LC-HRMS analysis showed that the active compound of charcoal in the caudal fins of Kerandang fish was hexadecanamide. The highest inhibition (IC_50_) of hyaluronidase was found in the ethyl acetate extract of fish caudal fins at a concentration of 4 mg/mL. We found that 15 mg/kg body weight of charcoal of fish caudal fins suppressed IgE expression in male mice.

**Conclusion:**

Our findings indicate that the charcoal of non-edible body parts of Kerandang and one of its constituent, hexadecanamide, may have strong antiallergic effects.

## Introduction

Many natural resources in Indonesia that are used in traditional medicine are now being studied for their potential application to the development of modern public health. However, despite the advancements of modern medicine and technology, traditional medicine remains a key component of public health in Indonesia, one that the government is still actively promoting [1]. The use of natural ingredients is advantageous compared to modern therapies; with only minimal side effects, natural materials are considered to be relatively safer than chemicals or synthetics on the market [2]. Allergy medication, for instance, is mostly reliant on synthetic drugs, such as antihistamines (AH), which have undesirable side effects. In an effort to minimize these unwanted side effects, people are starting to turn to more natural treatments. An allergic reaction or so-called hypersensitivity is an unnatural immunologic reaction in someone who has previously been sensitized with an antigen that causes an excessive immune reaction, which can manifest itself as inflammation or tissue damage. Rapid allergic reactions (i.e., anaphylactic reactions) are mainly mediated by immunoglobulin E (IgE). This reaction is characterized by a sudden response that occurs within minutes after the body is exposed to a source of antigens, thereby releasing mediators present in cells such as histamine, bradykinin, arachidonic acid, and prostaglandins. The release of these mediators causes allergic rhinitis, asthma, atopic dermatitis, skin flushing, and shortness of breath [3-6].

Allergens can be in the form of dust particles, plant dust, drugs, or food. The allergic mechanism is controlled by mast cells: When exposed to the allergen, they release the IgE antibody. IgE is also produced in large quantities when allergens attach to B lymphocyte cells [7,8]. The release of IgE then triggers degranulation and stimulates the release of histamine, leukotrienes, and other immune mediators, which triggers an allergic reaction. Like other immunoglobulins, IgE is produced by B- and plasma cells in response to an antigenic stimulus. The presence of interleukin (IL)-4 and IL-13 induces immunoglobulin class switching from other isotypes to IgE [9-11]. These two cytokines interact with receptors on the surface of B-cells to initiate a signaling cascade mediated by Janus kinase 3 and signal transducer and activator of transcription 6. A second signal is required for class switching to IgE to occur, and this involves CD40 on the B-cell interacting with the CD40 ligand on the T-cell. Once IgE is produced by allergen-specific B-cells, it is released into the circulation [12-14].

The Kerandang fish has recently gained significant attention in Central Kalimantan, as charcoal can be made from several of the inedible body parts of the fish, then used as a traditional medicine to treat hereditary allergic reactions. The body parts of the fish are burned into charcoal, and then smeared on itchy skin or on the bumps that arise on the skin after an allergic reaction. Many fish that inhabit peatlands possess various bioactive materials, which can be used for several medicinal purposes [15].

Thus far, the antiallergenic properties of charcoal from the Kerandang fish have not been studied. In this study, we attempt to characterize charcoal from the body parts of Kerandang fish (*Channa pleurophthalmus* Blkr) and identify its antiallergenic properties.

## Materials and Methods

### Ethical approval

This study was approved by Animal Care and Use Committee, Brawijaya University, Indonesia (approval no. 1075-KEP-UB).

### Study period

This study was conducted from September 2018 to September 2019. We obtained inedible parts of Kerandang fish, including the scalp, scales, dorsal fins, pectoral fins, ventral fins, anal fins, and caudal fins, from fishermen in Sebangau Kereng Bengkirai Lake, Central Kalimantan, Indonesia.

### Sample preparation

The fish samples were cleaned and dried for 2-3 days, then burned to charcoal using a normal oven with a maximum temperature of 200°C, until the resulting charcoal attained a mass of 500 g per sample. The extraction was done at a temperature of <4°C, using ethanol, ethyl acetate, and chloroform solvent, under the following protocol: 100 g of dry samples were added 500 mL of solvent, incubated for 24 h, and filtered through a vacuum filter. The filtrate was dried using a vacuum rotary evaporator. The crude extract was stored until further analysis [16].

### Liquid chromatography–high-resolution mass spectrometry (LC-HRMS)

Each extract was diluted in a solvent until reaching a volume of 1300 μL. All extracts were spun for 2 min and filtered using a 0.22 μm syringe filter. The samples were then inserted into the autosampler and injected into the LC-HRMS. The data were converted into a NetCDF format to ease data processing using mzCloud™ (HighChem LLC, Slovakia). MzCloud data processing consists of several steps, i.e., creating a chromatogram, reducing noise, identification based on molecular weight, and compiling data [17].

### *In vitro* antihyaluronidase test

The antihyaluronidase activity was examined with a slight modification from the original protocol [18,19]. The sample solution (1, 2, and 4 mg/mL) was dissolved in a mixed solvent (5% dimethyl sulfoxide in ethanol), while 50 μL bovine hyaluronidase (7900 units/mL) was dissolved in 0.1 M acetate buffer (pH 3.5), mixed with 100 μL of each sample solution, then incubated for 20 min at 37°C. Next, 100 μL of 12.5 mM calcium chloride was added to the reaction mixture and incubated for 20 min at 37°C. Bovine hyaluronidase activated by Ca^2+^ was reacted with 250 μL sodium hyaluronate (1.2 mg/mL) dissolved in 0.1 M acetate buffer (pH 3.5), then incubated at 37°C for 40 min. Next, 100 μL of 0.4 M sodium hydroxide and 100 μL of 0.4 M potassium borate were added to the reaction mixture and incubated in a bath of boiling water for 3 min. After cooling at room temperature, 1.5 μL of dimethylaminobenzaldehyde (DMAB) (4 g DMAB dissolved in 350 μL of 100% acetic acid and 50 μL of 10 M hydrochloric acid) was added to the reaction mixture, then incubated at 37°C for 20 min. Optical density (OD) in the reaction mixture was measured using a spectrophotometer at 585 nm. The percentage of inhibition was calculated using the following equation:

% Inhibitors = [(ODc − ODs) / ODc] × 100

Note: ODc is the OD of the control, ODs is the OD of the sample being tested. IC_50_ values causing 50% inhibition were determined using a linear regression analysis.

### *In vivo* IgE test

The highest charcoal antihyaluronidase activity was applied topically as a cream to ovalbumin (OVA)-induced allergy mouse for 7 days at three different treatment doses (10, 15, and 20 mg/kg); hydrocortisone cream was used as a positive control. The specific IgE was measured on day 8 after the application by collecting the blood serum using the Mouse OVA sIgE enzyme-linked immunosorbent assay marker kit.

### Statistical analysis

Data from the antihyaluronidase test and specific IgE levels were analyzed using a one-way analysis of variance using SPSS 23.0. LC-HRMS qualitative analyzed according to database mzCloud and compared with PubChem, Lipinski, and SwissADME bioinformatic method.

## Results and Discussion

### Antihyaluronidase test

**Figure-1:**
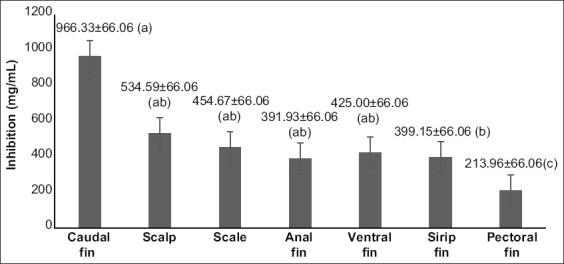
Hyaluronidase inhibition of charcoal extracts from several body parts of Kerandang fish that is not eaten.

**Figure-2:**
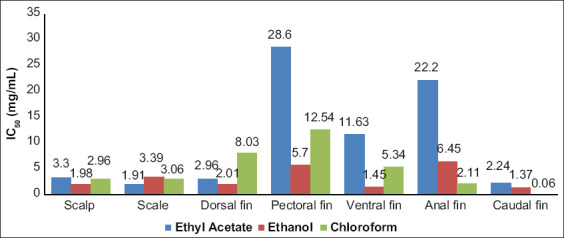
The antihyaluronidase activity (IC_50_) of charcoal extract of fish body that is not eaten at different solvent.

We measure the inhibition of hyaluronidase using charcoal extracts from inedible parts of the Kerandang fish body (Figure-1). The highest inhibition level was found in the caudal fins charcoal extract (966.33±66.06 mg/mL), while the lowest was in pectoral fins charcoal extract (213.96±66.06 mg/mL). Figure-2 shows that the IC_50_ value for the seven types of charcoal tested with three different solvents (chloroform, ethanol, and ethyl acetate). Data of IC_50_ of hyaluronidase activity were obtained from a linear regression analysis. IC_50_ values represent the extract concentration of samples needed to inhibit 50% of hyaluronidase activity, which determined by linear regression analysis [16]. The highest antihyaluronidase activity was found in caudal fins extracted with 4 mg/mL of ethyl acetate. This extract inhibited 50% of hyaluronidase activity at a concentration of 0.06 mg/mL. The lowest activity of antihyaluronidase was found in charcoal from Kerandang fish pectoral fins, which were extracted with 4 mg/mL chloroform (28.6 mg/mL) (Figure-2). Hyaluronidase is one of the essential enzymes involved in allergic and inflammatory reactions, playing a key role in mast cell degranulation [20-22]. This enzyme is also involved in the cancer migration, inflammation, and increased vascular system permeability in the extracellular matrix of connective tissues, affecting both organs (testes, spleen, skin, eyes, liver, kidneys, uterus, and placenta) and body fluids (tears, blood, and sperm) [23-25]. A previous study reported that hypo-allergies and anti-inflammatories were strong inhibitors of hyaluronidase activity [22], which led researchers to use hyaluronidase inhibitory activity as one of the parameters of hypo-allergenic testing [26,27].

### IgE levels

The IgE levels in male mice were increased after sensitization OVA (Table-1), which indicated an allergic reaction had occurred. The IgE levels in the bloodstream were very small, usually <1 U/mL (1 U=2.4 ng). IgE levels in normal individual serum ranged from 0.1 to 0.4 μg/mL. Individual IgE levels below 48 ng/mL are counter indicative of an allergic reaction, while IgE levels above 240 ng/mL indicate an allergy reaction [5]. Skin allergies are also induced by allergens, causing red, bumpy, scaly, itchy, or swollen skin. There are various types of allergies due to skin flora, culture, and diversity [27-29]. Understanding the role of IgE in allergic reactions has been a central part of research in this field.

**Table-1:** IgE specific results on male mouse group before sensitization ovalbumin and after sensitization ovalbumin.

Group	Result specific IgE (ng/mL)	

	Pre-sensitization	After sensitization
I	95.7	434.6
	91.2	412.3
	89.9	430.9
II	92.1	591.2
	101.1	475.8
	98.7	466.6
III	102.3	1121.8
	99.1	908.7
	102.3	1147.7
IV	99.2	647.3
	112.4	789.6
	90.9	709.4
V	96.7	749.1
	102.9	736.2
	92.9	549.9

Group I=Negative control group, Group II=Positive control group, Group III=Treatment 1 group, Group IV=Treatment 2 group, Group V=Treatment 3 group, IgE: Immunoglobulin E

**Figure-3:**
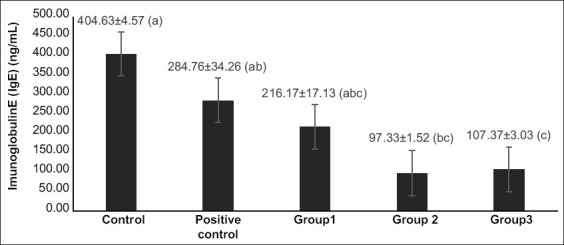
Specific immunoglobulin E levels in male mouse group after giving charcoal doses from Kerandang fish caudal fins. Note: Control: Mice without treatment; Positive control group: hydrocortisone cream; Group 1: Charcoal dose group of 10 mg/kg body weight (BW); Group 2: Charcoal dose group as much as 15 mg/kg BW; Group 3: Charcoal dose group as much as 20 mg/kg BW.

The average level of IgE in control mice was 404.63±4.57 ng/mL (Figure-3). In the group given hydrocortisone cream, the average specific lgE level was 284.76±34.26 ng/mL. The group given a charcoal dose of 10 mg/kg body weight (BW) (Group 1) had an average specific level of IgE of 216.17±17.13 ng/mL. Group 2 was given a charcoal dose of 15 mg/kg BW and had an average specific IgE level of 97.33±1.52 ng/mL, while Group 3 received a dose of 20 mg/kg BW had an average level 107.37±3.0 ng/mL. Of the five treatment groups, Group 2 had the lowest average specific IgE level, suggesting that the treatment dose was able to reduce specific IgE levels in the blood of male mice, while the control group had the highest level of specific IgE, suggesting the control group did not decrease IgE levels. In the three treatment groups with tail fin charcoal doses, the group treated with 15 mg/kg BW had the lowest average specific lgE level, while 10 mg/kg BW induced the highest average specific lgE levels. This result suggests that the treatment of a 10 mg/kg BW dose did not significantly reduce IgE levels in male mice. The treatment that caused a significant impact was 15 mg/kg BW dose (Group 2), which induced a decrease in IgE levels in male mice.

Histamine plays a key role in the pathogenesis of allergies through the regulation of differentiation of CD4+ Th-cell lymphocytes [30]. The mechanism through which AH operates is by blocking histamine receptors to incoming histamines or by expelling histamine already in the receptor [31]. By inhibiting mast cell degranulation, the secretion of vasoactive amines, such as histamine, lipid mediators, and cytokines playing a role in the inflammatory process in allergic reactions, is also reduced. AH have long been prescribed for atopic dermatitis as adjunctive therapy with topical agents that can block the action of histamines on the skin [32].

Topical nasal AHs such as azelastine are also available and recommended forrunny nose due to allergic effects. To increase the efficacy of oral AHs in allergic rhinitis for those who continue to exhibit symptoms, the preferred topical therapy is a corticosteroid nasal spray. These sprays should be considered a first-line treatment in moderate to severe allergic rhinitis [33,34].

Treatment for allergic reactions takes several forms: One can increase IgE levels so incoming antigens can be destroyed through the complement system; others provide the patients with AHs, reduce IgE levels to inhibit the binding between antigens with IgE, prevent the entry of antigens into the body, or again inhibit mastocyte degranulation to prevent the release of chemical mediators, which stimulate the allergic reaction [35-37]. In other cases, antiallergenic drugs work by inhibiting the degranulation of Rat Basophilic Leukemia-2H3 cells, using β-hexosaminidase as a biomarker, actinomycete Nesterenkonia flava, at the epidermis environment interface [38,39].

### LC-HRMS analysis

**Figure-4:**
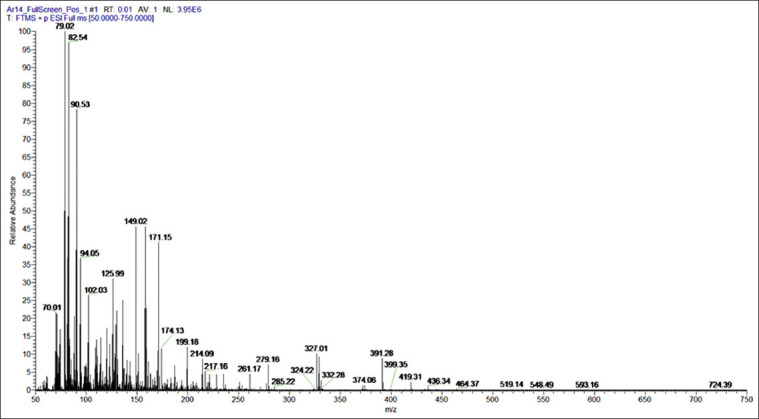
Liquid chromatography–high-resolution mass spectrometry chromatogram results.

**Figure-5:**
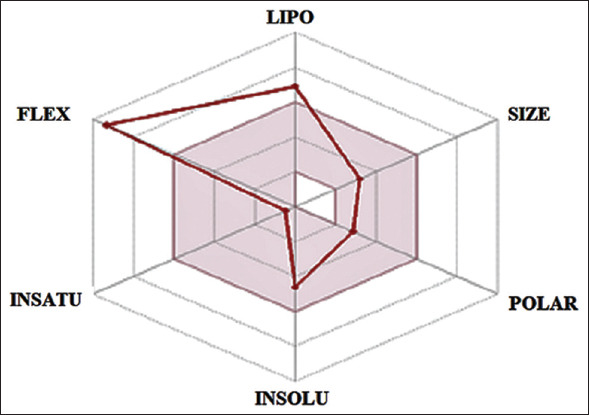
Screening availability hexadecanamide using SwissADME. The colored zone is the suitable physicochemical space for oral bioavailability. Notes: LIP (Lipophility): -0.7 < XLOGP3 < +50; SIZE: 150 g/mol < MV < 500 g/mol; POLAR (Polarity): 20A^2^ < TPSA < 130A^2^; INSOLU (insolubility): 0 < Log 5 (ESOL) < 6; INSATU (Unsaturation): 0.25 < Fraction Csp3 < 1; FLEX (Flexibility): 0 < Number rotatable bonds < 9.

The identification of charcoal ethyl acetate extract from Kerandang fish caudal fins using LC-HRMS is shown in Figure-4. The spectral results show that the ethyl acetate extract of the Kerandang fish caudal fins obtained eight peaks at a retention time of 1.06; 12.12; 12.64; 16.57; 17.74; 18.08; 20.10, and 26.39 min. LC-HRMS data were analyzed using the mzCloud MS/MS library data software and identification of the structure of chemical compounds detected in the LC-HRMS with the PubChem, Lipinski, and SwissADME online database. The ethyl acetate extract of charcoal made from Kerandang fish caudal fins analyzed using LC-HRMS Best Match produced 49 compounds, which were identified based on their mass spectrum and mzCloud values. The highest value recorded (>90) was identified as the hexadecanamide compound. Hexadecanamide is a derivative of palmitic acid also found in palmitoylethanolamide (PEA). It is also sometimes referred to as n-(2-hydroxyethyl) hexadecanamide, n-hexadecanoylethanolamine, palmidrol PEA, palmitylethanolamide, PEA, n-(2-hydroxyethyl) hexadecanamide, or again n-(2-hydroxyethyl)-hexadecanamide palmid. The chemical formula for hexadecanamide formula is C_16_H_33_NO [39]. According to theLipinski method, there are five criteria of drugs: Molecular mass <500 Da, high lipophilicity (LogP <5), <5 hydrogen bond donors, <10 hydrogen bond acceptors, and molar refractivity from40 to 130 m^3^mol^−1^ [40]. Comparison results based on 3D_CID conformer 3008318 PubChem on hexadecanamide compounds are as follows: A molecular mass of 255.00 a logP of 4.95, two hydrogen bond donors, two hydrogen bond acceptors, and a molar reactivity of 79.51. Therefore, hexadecanamide meets all five drug criteria required by the Lipinski method. Hexadecanamide has been shown to have anti-inflammatory, anti-nociceptive, nervous, anticonvulsant, and antifungal properties [41-43]. Based on screening availability using SwissADME (Figure-5), it was suggested that hexadecanamide has a high potential as an anti-allergenic drug based on its bioavailability, lipophilicity, size, polarity, solubility, saturation, and flexibility.

## Conclusion

Our findings indicate that the charcoal of non-edible body parts of Kerandang and one of its constituent, hexadecanamide, may have strong antiallergic effects. More methodological work is needed on how to determine the effect inedible Kerandang fish body part on other parameter related to skin allergy.

## Authors’ Contributions

AA designed the research and wrote the manuscript. ES helped in experimental method. BBS collected and analyzed the data and HH revised the manuscript. All authors read and approved the final manuscript.
